# The Insulin-like Growth Factor Signalling Pathway in Cardiac Development and Regeneration

**DOI:** 10.3390/ijms23010234

**Published:** 2021-12-26

**Authors:** Sandra Díaz del Moral, Maha Benaouicha, Ramón Muñoz-Chápuli, Rita Carmona

**Affiliations:** 1 Institute of Biomedical Research of Málaga (IBIMA), Department of Animal Biology, Andalusian Center for Nanomedicine and Biotechnology (BIONAND), Faculty of Science, University of Málaga, 29071 Malaga, Spain; sandradiaz@uma.es (S.D.d.M.); benaouicha.maha@gmail.com (M.B.); chapuli@uma.es (R.M.-C.); 2 Department of Human Anatomy and Embryology, Legal Medicine and History of Medicine, Faculty of Medicine, University of Málaga, 29071 Malaga, Spain

**Keywords:** insulin-like growth factors, insulin-like growth factor receptors, heart development, myocardial proliferation

## Abstract

Insulin and Insulin-like growth factors (IGFs) perform key roles during embryonic development, regulating processes of cell proliferation and survival. The IGF signalling pathway comprises two IGFs (IGF1, IGF2), two IGF receptors (IGFR1, IGFR2), and six IGF binding proteins (IGFBPs) that regulate IGF transport and availability. The IGF signalling pathway is essential for cardiac development. IGF2 is the primary mitogen inducing ventricular cardiomyocyte proliferation and morphogenesis of the compact myocardial wall. Conditional deletion of the *Igf1r* and the insulin receptor (*Insr*) genes in the myocardium results in decreased cardiomyocyte proliferation and ventricular wall hypoplasia. The significance of the IGF signalling pathway during embryonic development has led to consider it as a candidate for adult cardiac repair and regeneration. In fact, paracrine IGF2 plays a key role in the transient regenerative ability of the newborn mouse heart. We aimed to review the current knowledge about the role played by the IGF signalling pathway during cardiac development and also the clinical potential of recapitulating this developmental axis in regeneration of the adult heart.

## 1. Introduction

The heart is the first functional organ to develop. It starts to beat and pump blood at around 21–22 days of development in the human foetus and 8 days in the mouse embryo [1]. The heart undergoes rapid growth in order to meet the increasing metabolic demands of the developing embryo. During embryonic and foetal life, this growth is mainly accomplished through hyperplasia, an increase in the number of myocardial cells. After birth, myocardial cells soon lose their proliferating potential. Thus, postnatal growth of the heart is due to hypertrophy of cardiomyocytes and proliferation of cardiac non-muscle cells. The hypertrophic growth leads to a 30- to 40-fold increase in volume of individual myocardial cells [2].

The insulin-like growth factor 1 (IGF1) signaling pathway is a highly conserved system regulating multiple cellular processes including proliferation, differentiation, metabolism and glucose homeostasis [3,4]. During development, elements of the insulin growth factor (IGF) signalling pathway are essential for the process of cardiac hyperplasia and the resulting cardiac growth. Among these elements, IGF2 has been identified as the most important mitogen for cardiomyocytes. IGF2 is secreted by different cell types, and it promotes cardiomyocyte proliferation through pathways mediated by different receptors. Other modulating elements of this pathway, such as the IGF binding proteins (IGFBPs) are involved in the regulation of the process. In this review, we will summarize the knowledge about the functions played by the elements of the IGF signalling pathway in cardiac development. We will also review the role played by the IGF signalling pathway in cardiac regeneration and myocardial differentiation of stem cells.

## 2. Insulin-like Growth Factors in Cardiac Development

**Figure 1:**
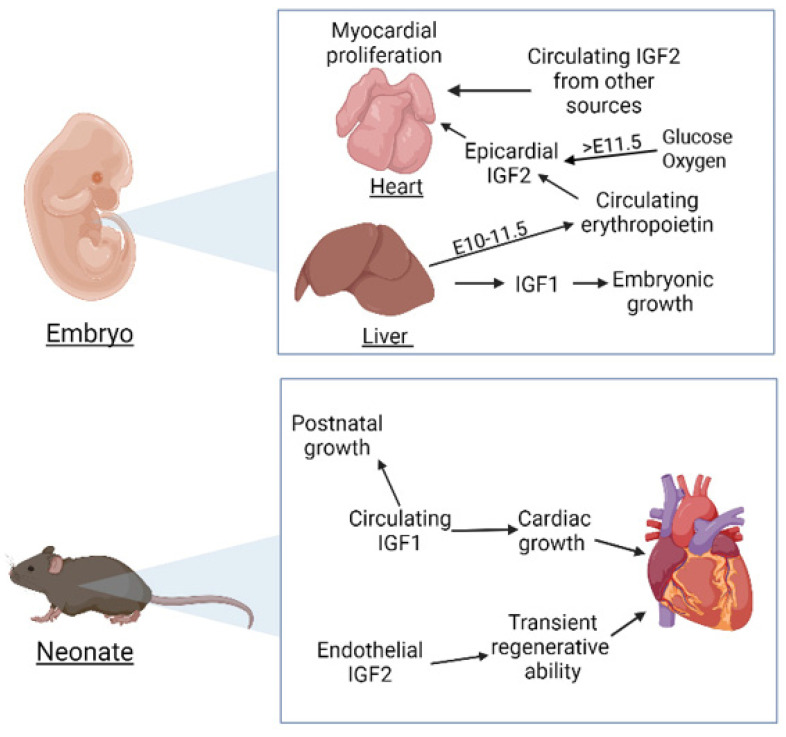
Main functions of the IGF signalling system in cardiac development and in the heart of neonate mice. Both cardiomyocyte proliferation and postnatal cardiac growth are regulated by this system, where IGF1 and IGF2 play different roles.

IGF1 is a polypeptide composed of 70 residues organized in four domains, named A–D. Proinsulin includes the A-C domains while mature insulin is composed only of the A and B domains. Circulating IGF1 is mainly secreted by the liver and regulated by growth hormone, but many tissues can express IGF1 in an autocrine/paracrine manner [5]. Loss of function of the *Igf1* gene provokes growth retardation and perinatal lethality [6,7]. IGF1 also plays an important role regulating the postnatal growth of the heart and activating both canonical and non-canonical signalling pathways in the adult heart [8,9] (Figure 1). It has been involved also in cardiac aging [10]. However, its role during cardiac development seems to be less relevant than that played by IGF2 (see below), but a study of IGF1 knockdown in chick embryos and in cultured cardiomyocytes showed increased oxidative stress and metabolic dysfunction [11]. On the other hand, IGF1 played an anti-apoptotic protective effect in the cardiomyocytes of tbx5-deficient zebrafish embryos, reducing the incidence of the cardiac phenotypic anomalies [12].

*Igf2* is an imprinted gene, expressed from the paternally allele in most cases. It encodes a 67-amino acid single-chain secreted protein also organized in four domains (A–D) and essential for foetal growth and development [13,14] (Figure 1). IGF2 knockout mice show generalized growth retardation during development [15]. However, the postnatal growth is not affected by the IGF2 loss of function. In fact, levels of circulating IGF2 decrease dramatically in mice after birth. In contrast, IGF1 ablation provokes postnatal growth retardation and other defects in adults [16,17].

IGF2 is the main mitogen for cardiomyocytes during foetal growth [18,19,20]. The mRNA levels of IGF2 are much higher in the embryonic ventricular tissue than the levels of IGF1. The expression of IGF2 decreases after the birth, and they become unnoticeable in young adults. IGF2 treatment of neonatal cardiomyocytes induces hypertrophy but not proliferation [20].

**Figure 2:**
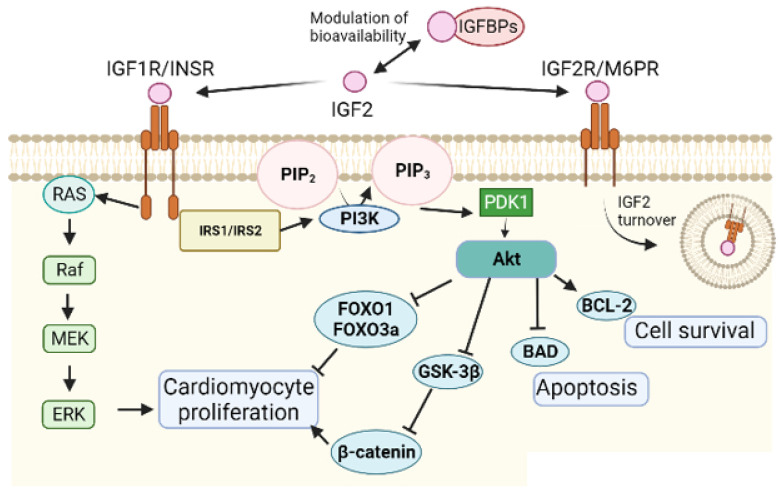
The insulin growth factor signalling pathway in myocardial development. The relative importance of IGFR1 and INSR in the transduction of the mitogenic IGF2 signal has been debated, but IGF1R seems to be the main receptor in the embryonic cardiomyocytes. IGFBPs modulate bioavailability of IGF2. IGF2R is a negative regulator of the system by receptor-mediated endocytosis of IGF2. The main transduction pathways activated by IGF1R are the ERK/MAPK and the PI3K/Akt cascades, leading to proliferation and survival of the cardiomyocytes.

IGF2 promotes foetal cardiomyocyte proliferation through the tyrosine kinase receptors IGF1R and INSR as described below (Figure 2). The transduction of the IGF signal leads to activation of the ERK/MAPK and the PI3K/Akt pathways [19,20,21,22]. Activated Akt leads to cytoplasmic localization and inactivation of FOXO factors, negative regulators of myocardial proliferation [23]. IGF1 does not have a similar proliferative activity on the developing myocardium. However, overproduction of this factor induced by an excess of growth hormone in human acromegaly leads, in many cases, to cardiomyopathy characterized by concentric hypertrophy as well as anomalies in cardiac rhythm and valves [24]. Elevated IGF1 levels in serum are diagnostic of acromegaly [25].

The cardiomyocytes also express IGFs [17], but the main source of IGFs during cardiac development is the epicardium. Both, IGF1 and IGF2 are expressed by the epicardium between E11.5 and E17.5. The epicardial secretion of IGF2 is required for cardiomyocytes proliferation until the establishment of the coronary circulation, and this signalling involves the ERK pathway [18]. The epicardial source of IGF2 is first induced by circulating erythropoietin (EPO) (Figure 1). In fact, when cultured epicardial cells are treated with EPO, expression of IGF2 is upregulated, but the same effect is not induced by retinoic acid (RA). IGF2 expression and ventricular cardiomyocyte proliferation is restored even in raldh2-deficient hearts by EPO treatment [26]. The authors propose that liver is the origin of the RA-induced expression of EPO necessary for IGF2 induction in the epicardium. After this EPO-dependent stage (E10–E11.5), epicardial IGF2 expression becomes regulated by the influx of glucose and oxygen associated with placental transport function [27,28].

## 3. Insulin and IGF Receptors in Cardiac Development

The insulin receptor (INSR) and the insulin-like growth factor 1 receptor (IGF1R) are heterotetramers composed of two α and two β subunits (Figure 2). The α subunits are transmembrane ligand-binding polypeptides while the β subunits are intracellular and contain the tyrosine kinase domains. The high degree of homology between the subunits of INSR and IGF1R allows for the formation of hybrid receptors [29,30].

When activated, both receptors phosphorylate the same targets, including members of the insulin receptor substrate (IRS) proteins, and activate the same signalling pathways, basically the phosphatidylinositol 3-kinase (PI3K)/Akt and the mitogen activated protein kinases (MAPK) [22]. Recently, evidence of other non-classical functions of the INSR and the IGF1R have been proposed, including transcriptional regulation in the nucleus. Strikingly, IGF1R can bind and activate its own gene promoter, suggesting some kind of autoregulatory mechanism [31].

IGF1R is the main receptor mediating IGF signalling in the heart. However, the relative importance of IGFR1 and INSR in the transduction of the mitogenic IGF signals has been debated. Single IGFR1 or INSR loss of function in cardiomyocytes has no effect on viability. Double knockout of both receptors in cardiomyocytes (MI2RKO model) is lethal during the first month of life. Young mice show dilated cardiomyopathy, metabolic alterations and anomalous expression of contractile proteins. When only INSR is deleted (CIRKO model) mice survive but they show lower performance at 6 months of age (the phenotype is described below). Deletion of INSR in mice hemizygous for IGFR1 slightly increases the postnatal mortality while the presence of INSR, either in homozygosis or hemizygosis, compensates the loss of function of IGFR1 [22]. According to these findings, INSR would not be critical for cardiac development, but it could mediate the mitogenic response in an IGFR1 loss of function. However, other studies have found that INSR is much less activated than IGFR1 by IGF2 between E10 and E13, and INSR cannot compensate the IGFR1 loss of function. Thus, IGFR1 would be the main IGF2 receptor involved in the morphogenesis of the ventricular wall [32].

The CIRKO model was developed through conditional deletion of the insulin receptor in cardiomyocytes. The cardiac size of the CIRKO mice is reduced by 20–30%. The cardiomyocytes are smaller and show persistent expression of the foetal β-MHC isoform. Metabolic changes were also observed, including increased rates of glucose uptake and glycolysis, while the use of fatty acids as energy source was reduced. A similar phenotype is exhibited by the MIRKO model (deletion of the insulin receptor in both skeletal muscle and cardiac muscle). These mice are dyslipidemic and they also show the reduction in heart size with little impact on cardiac function [33,34]. Thus, insulin signalling is required for regulation of cardiac size, myosin isoform expression and cardiac substrate utilization [34].

As stated above, IGF2 promotes proliferation through the tyrosine kinase receptors IGF1R and INSR [20]. This pathway is regulated by YAP, an effector of the Hippo pathway. Cardiac conditional deletion of YAP decreases cardiomyocyte proliferation and provokes myocardial hypoplasia leading to embryonic lethality at the stage E10.5 [35]. On the other hand, constitutive activity of YAP induces increased myocardial proliferation and cardiac size. The activation of the IGF signalling pathway by YAP is mediated by inhibition of GSK3 and activation on the canonical β-catenin pathway, inducing cardiomyocyte proliferation [35,36].

Modulation of the IGF signalling system can be an adaptive response to growth restriction during development. The levels of IGF2, IGF1R and IGF2R mRNA are increased in the heart of lamb foetuses with growth restriction induced by placental insufficiency. On the other hand, young lambs with low birth weights show greater left ventricle/body weight ratio and increased cardiac expression of IGF2 and IGF2R mRNA [37]. These authors conclude that altered IGF signalling due to growth restriction in foetal life and accelerated growth in childhood can be related with the greater risk of coronary heart disease associated to these developmental anomalies.

The function of the insulin-like growth factor 2 receptor (IGF2R) is different to that of IGF1R. IGF2R lacks tyrosine kinase domains and it is involved in IGF2 turnover by receptor-mediated endocytosis, regulating negatively the IGF pathway [38] (Figure 2). This transmembrane glycoprotein is also known as the mannose-6-phosphate (M6P) receptor, binding M6P-bearing lysosomal enzymes and other ligands, such as transforming growth factor-β or leukemia inhibitory factor [39]. In this way, IGFR2/M6P receptor plays an important role in the intracellular trafficking of lysosomal enzymes.

The imprinted *Igf2r* gene is normally expressed from the maternal allele. When this maternally derived gene is disrupted, IGF2 levels increase leading to excessive body size (135% of normal weight at birth), organomegalia and cardiac anomalies. The heart volume is four times larger at E18.5 as compared with wild-type embryos, showing mural thickening of the left ventricle. However, the cardiomyocytes are not hypertrophied. The mutation is usually lethal at birth. These mutants can be rescued by a loss of function of IGF2 or IGF1R. The Igf1r/Igf2r double mutants show normal embryonic development, only differing from controls in the postnatal growth pattern [38].

Embryonic epicardial cells also express IGFR1 and they are responsive to exogenous IGF signalling. In fact, pharmacological inhibition of IGF receptors decreases FAK phosphorilation and epicardial proliferation [40].

## 4. MicroRNA Regulation of the IGF Signalling Pathway

MicroRNAs can regulate the IGF pathway during cardiac development. Mice lacking miR-1 show severe cardiac defects and increased lethality by weaning [41]. A half of the embryos showed ventricular septal defect and surviving adults exhibited electrocardiographic anomalies. Both IGF1 and IGF1R are targets of miR-1 [42]. IGF1R is also a target of other miRNA highly expressed in the postnatal cardiomyocyte, miR-378. In fact, IGF1R is downregulated postnatally, when the levels of miR-378 increase. Interestingly, IGF1 inhibits the expression of miR-378. These findings suggest an interaction between miR-378, IGF1R, and IGF-1 which could be involved in the postnatal remodelling of the heart [43].

On the other hand, miR-133 is involved in the proliferation, differentiation, survival, hypertrophic growth, and electrical conduction of cardiac cells [44]. IGF1R is a target of miR-133 during skeletal myogenesis and cardiac hypertrophy [45,46]. However, the correlations between the expression of miR-1, miR-133 and the IGF pathway has been more extensively studied in relation with cardiac hypertrophy and heart failure than in the role that they could play in cardiac development (reviewed in [4,47,48]).

## 5. The IGF-Binding Proteins Family

Insulin is found free in the blood, but the IGFs circulate bound to IGF-binding proteins (IGFBPs). Free IGFs in circulation have a short half-life (10–12 min). IGFBPs prolong this half-life and regulate the movement of IGFs into tissues and their activity [49]. Seven IGFBPs have been described in mammals. They have different binding affinities and different spatial and temporal patterns of tissue expression [13,50]. Depending on the level of expression and the cellular context, they can stimulate or inhibit IGF action. So, IGFBP3 is the major carrier of IGFs in blood, potentiating their activity and inhibiting them when it is overexpressed. IGFBP4, instead, is always inhibitory and IGFBP6 binds specifically IGF2, inhibiting its action [13,51].

Recent findings have shown the role played by IGFBPs in cardiac development, acting as modulators of the IGFs. Zebrafish with overexpression of Igfbp2a in the developing heart resulted in a significant 78% reduction in its relative size [52]. IGFBP3 is upregulated in the endocardium and endothelium of mouse embryos deficient in Ino80, a chromatin remodeller gene. This excess decreases cardiomyocyte proliferation and contribute to non-compaction cardiomyopathy [53]. *Igfbp5* is a putative target gene of SHOX2, a transcription factor involved in the development of the cardiac pacemaker [54]. On the other hand, IGFBP4 enhanced cardiomyocyte differentiation from embryonic and induced pluripotent stem cells and also promoted their proliferation [55,56]. This effect is mediated by inhibition of β-catenin signalling and it is independent of the IGF axis [57]. This is an example of the roles played by IGFBPs beyond modulation of IGF activity [50].

## 6. The Insulin Receptor Substrate Family

The IRS proteins are adaptor proteins that serve as scaffolds to organize signalling complexes and initiate intracellular signalling pathways. They are involved in the transmission of signals from the insulin and IGF1 receptors. They are ubiquitously expressed and mediate insulin-dependent mitogenesis and regulation of glucose metabolism in most cell types [58]. IRS1 is a phosphoprotein containing a phosphotyrosine binding domain. IRS2 is also phosphorylated by the activated insulin receptor. Other members of the family have a more restricted tissue expression or are specific of rodents and humans (reviewed in [59]). Double IRS1-2 loss of function in cardiomyocytes lead to cardiac dilatation, fibrosis and progressive heart failure, causing the death of the mice between 6–8 months [60].

## 7. Alternative IGF1R Ligands

Other ligands can activate the IGF signalling pathway. The product of the *Dipk2a* gene is called the hypoxia and Akt-induced stem cell factor (HASF). This novel paracrine factor stimulates cardiomyocyte proliferation through activation of the PI3K/Akt signalling cascade [61]. Treatment of primary adult rat cardiomyocytes with HASF inhibits apoptosis in culture and direct injection of HASF protein in the heart after myocardial infarction has a protective effect, reducing fibrosis and improved cardiac function as compared with controls [62]. The HASF receptor on the cardiomyocytes was recently identified as the IGF1R. In fact, treatment of neonatal cardiomyocytes with HASF induces phosphorylation of IGF1R. When this receptor is pharmacologically inhibited, HASF-mediated ERK activation and cell proliferation are blocked. However, knockdown of IGF2R or INSR has no effect. Thus, HASF acts as a novel ligand for IGFR1 [63].

## 8. IGF Signalling in Differentiation of Cardiomyocytes from Stem Cells

Treatment of murine embryonic stem cells with insulin, IGF1 or IGF2 enhances mesoderm differentiation and increases the number of Nkx2.5 expressing cardiac progenitor cells [64]. Differently to mouse ESC-derived cardiomyocytes, cardiomyocytes derived from human ESC are highly proliferative in serum-free media, and this proliferation is dependent on the phosphatidylinositol 3-kinase/Akt signalling pathway. Inhibition of the IGFR1 by blocking antibodies decreases proliferation, while treatment with IGF1 or IGF2 increases growth in a dose-dependent manner. Thus, the axis IGF/PI3K/Akt seems to be relevant for proliferation of hESC-derived cardiomyocytes [65]. However, the ERK pathway has been associated to the foetal cardiomyocyte proliferation in other studies as stated above [19].

Partenogenetic stem cells (PSC) are an interesting alternative for allogeneic cell therapies due to the haploidentity of major histocompatibility complexes [66]). Since *Igf2* is a paternally imprinted gene, as stated above, myocardial differentiation of PSC is impaired due to the lack of IGF2 expression. However, induced expression of IGF2 in PSC accelerated PSC into the cardiac lineage and promoted cardiomyocyte maturation, improving the regenerative potential of these cells in infarcted hearts [67].

## 9. IGF Signalling in Cardiac Regeneration

Differently to the adult mammalian heart, adult zebrafish heart regenerates after injury. IGF signalling is important for this process of regeneration, which involves cardiomyocyte proliferation. Igf2b expression increases in the zebrafish heart after resection of the ventricular apex, coinciding with the re-entry of cardiomyocytes in the cell cycle. Zebrafish carrying a dominant negative *Igf1r* or treated with an *Igf1r* inhibitor during development showed fewer cardiomyocytes and defective heart development, and the adult inhibition of *Igf1r* impaired the regenerative process reducing the proliferative ability of the cardiomyocytes [68].

Newborn mouse heart can also regenerate after injury, but this ability is lost one week after birth. Again, IGF2 secreted by endothelium and endocardium plays a key role in this transient regenerative potential. Ablation of IGF2 expression abolished cardiomyocyte proliferation induced by injury one day after birth [69]. Thus IGF2 is a paracrine factor involved in the early regenerative ability of the mouse heart.

Cardiomyocytes of newborn mice proliferate in the few days after birth. This proliferation can be stimulated with thyroid hormone (T3) treatment, which increases expression of IGF1 and IGF1R activating ERK1/2 signalling [70]. This response to T3 is maintained at P8, but only in the apex of the left ventricle [71]. The lack of response in the base of the left ventricle is due to the expression of the nuclear phospho-ERK1/2-specific dual-specificity phosphatase, DUSP5. The expression of DUSP5 progresses from the base to the apex of the left ventricle between P7 and P14, inhibiting the proliferative ERK1/2 signalling.

Despite the lack of regenerative potential of the adult mouse heart after injury, a role for IGF1 has been shown in the promotion of cardiac progenitor cell (CPC) survival in obese mice. The higher number of cKit^+^/CD45^−^ CPC in IGF1-treated obese mice compared with controls is associated to a significant improvement of the cardiomyopathy provoked by Western diet-induced obesity [72].

YAP is a critical regulator of cardiomyocyte proliferation during cardiac development through activation of the IGF pathway, as stated above [35]. Furthermore, neonatal heart regeneration is blocked if YAP1 is deleted in cardiomyocytes [73]. Thus, postnatal activation of YAP1 in postnatal cardiomyocytes has been proposed as a useful strategy to stimulate cardiomyocyte expansion in therapeutic myocardial regeneration [36,73].

## 10. Conclusions and Future Directions

Cardiovascular diseases are the leading cause of death globally. The precise knowledge of the developmental programs of cardiac development can contribute to the treatment of these diseases either by early detection and eventual correction of congenital anomalies or by recapitulation of some of these developmental programs to regenerate and repair the failing heart. The role played by the IGF signalling pathways in myocardial proliferation during the foetal life is relatively well known. However, some uncertainties still persist, for example, how the IGFBPs modulate the pathway in the different stages of cardiac development. We do not know if the role played in adult cardiomyocytes by miRNAs such as miR-1, miR-133 or miR-378 correlates with regulatory functions of the IGF pathway in the developing myocardium. The recent description of non-classical functions of the INSR and the IGF1R, including transcriptional activity [31], raises the question of the involvement of these novel functions in cardiac development. Finally, the translational potential of a better knowledge of the IGF signalling system has been highlighted by the recent experimental evidence. This system participates in the adaptive response of the heart to foetal growth restriction, the transient regenerative potential of neonatal cardiomyocytes, the survival of cardiac progenitor cells and the proliferation of stem cell-derived cardiomyocytes. Indeed, the IGF-based therapies are currently being applied in the treatment of the myocardial infarction and other cardiomyopathies, although this issue is out of the scope of our review. In summary, the fine tuning of the IGF signalling system can provide useful strategies in the fight against cardiovascular diseases.

## Data Availability

Not applicable.

## Abbreviations

CIRKO	Cardiomyocyte-restricted deletion of insulin receptors
CPC	Cardiac progenitor cell
DIPK2A	Divergent protein kinase domain 2A
DUSP5	Dual Specificity Phosphatase 5
E	Embryonic day
EPO	Erythropoietin
ERK	extracellular signal-regulated kinase 1
ESC	Embryonic stem cells
FAK	Focal adhesion kinase
GSK3	glycogen synthase kinase 3
HASF	Hypoxia and Akt-induced stem cell factor
hESC	Human embryonic stem cells
IGF	Insulin-like growth factors
*Igf*	Insulin-like growth factors gene
IGFBP	Insulin-like growth factors binding proteins
IGFR	Insulin-like growth factor receptor
INSR	Insulin receptor
*Insr*	Insulin receptor gene
IRS	Insulin receptor substrate
M6P	Mannose-6-phosphate
MAPK	Mitogen activated protein kinases
MI2RKO	Double knockout of both receptors in cardiomyocytes
miR	MicroRNAs
miRNA	MicroRNAs
mRNA	Messenger RNA
PI3K	phosphatidylinositol 3-kinase
P	Postnatal day
RA	Retinoic acid
*Raldh2*	Retinaldehyde dehydrogenase 2 gene
T3	Thyroid hormone
YAP	Yesassociated protein
